# Factors associated with non-uptake of measles-rubella vaccine second dose among children under five years in Mtwara district council, Tanzania, 2017

**DOI:** 10.11604/pamj.2019.33.67.17055

**Published:** 2019-05-29

**Authors:** Richard Magodi, Elia John Mmbaga, Julius Massaga, Dafrosa Lyimo, Alex Mphuru, Ahmed Abade

**Affiliations:** 1 Health Department, Ilala Municipal council, P.O.BOX 20950, Dar-es-Salaam, Tanzania; 2 Field Epidemiology and Laboratory Training Programme, Dar-es-Salaam, Tanzania; 3 Department of Epidemiology and Biostatistics, Muhimbili University of Health and Allied Sciences, P.O.BOX 65001, Dar-es-Salaam, Tanzania; 4 National Institute of Medical Research, Ministry of Health, Community Development, Gender, Elderly and Children, P.O.BOX 9083, Dar-es-Salaam, Tanzania; 5 Immunization and Vaccine Development, Ministry of Health, Community Development, Gender, Elderly and Children, P.O.BOX 9083, Dar-es-Salaam, Tanzania

**Keywords:** Measles-rubella, immunization, vaccination, Tanzania

## Abstract

**Introduction:**

in 2014, Tanzania introduced the combined measles-rubella vaccine in the routine immunization schedule. Two doses of measles-rubella vaccine (MR1 and MR2) are recommended at 9 and 18 months, respectively. In 2015, MR2 coverage among eligible 18-month-old children in Tanzania was only 57%, lower than the WHO-recommended coverage (95%). During the same period Mtwara District Council (MDC) reported a coverage of 52% which is lower than the nation average. We determined factors associated with non-uptake of MR2 among children in MDC Tanzania.

**Methods:**

we conducted a community-based cross-sectional survey using cluster sampling during January - April 2017 in MDC. Caretakers of children born during January 2014 - January 2015 and residing in MDC for the past three years were recruited. We interviewed participants and reviewed vaccination cards. Logistic regression modeling was employed to identify independent factors associated with uptake of MR2.

**Results:**

of 1,000 children assessed, 558 (55.8%) were unvaccinated with MR2. Factors independently associated with non-uptake of MR2 included the caretaker being unaware of the ages for MR1 and MR2 administration [aOR=3.50; 95%CI 1.98-6.21; p<0.001], having MR2 vaccination services offered at the local vaccination station fewer than three days per week [aOR=1.50; 95%CI 1.42-5.59; p<0.001], not having the vaccine available during vaccination days [aOR=3.38; 95%CI 1.08-10.61; p<0.01], unwillingness of health workers to open multi-dose vaccine vials for a single child [aOR=3.80; 95% CI 2.12-6.79; p<0.001], and long waiting times for vaccination services [aOR=1.80; 95% CI 1.08-3.00; p<0.01].

**Conclusion:**

more than half the children under five years in MDC were not vaccinated with MR2. Lack of caretaker knowledge about appropriate vaccination age, unavailability of vaccine, having insufficient numbers of children waiting to warrant multidose vial use, and long clinic waiting times were associated with MR2 non-uptake. The community should receive education about MR vaccine; we recommend thorough screening of children?s vaccination status at each clinic visit and provision of vaccine whenever possible. Vaccine distribution should be improved in MDC.

## Introduction

Measles and rubella are viral diseases that remain important public health problems in both developed and developing countries. Measles caused 134,200 deaths globally in 2015, mostly among children under the age of five years [1]. WHO recommends that, in order for children to receive full protection against measles infection, they receive two doses of measles-containing vaccine at ages 9-12 months and 15-18 months. Although vaccination is vital in the drive to decrease global childhood mortality and morbidity due to measles, challenges remain in ensuring wide vaccine coverage. In October 2014, Tanzania introduced the two-dose combined measles-rubella (MR) vaccine into the national routine immunization schedule, to be given at 9 months (MR1) and 18 months (MR2) of age. Although Tanzania has been able to achieve high vaccination coverage rates for almost all vaccines being offered, MR2 vaccination coverage has lagged behind. Overall coverage for MR2 in 2014 and 2015 was 30% and 57%, respectively, among children aged 18 months. However, the reasons for poor uptake of MR2 remain unknown. Therefore, we conducted a study to identify factors associated with non-uptake of MR2 among children born during January 2014 to January 2015 in a district with poor coverage, Mtwara District Council. The findings are intended to guide the council health management team of Mtwara District Council to develop population-specific interventions to improve vaccination coverage.

## Methods

### Study area

Mtwara District Council is one of seven councils in Mtwara region in Tanzania. According to the 2012 Tanzania National Census, the population was 204,770. The council is divided into six divisions, 21 wards, and 101 villages. Mtwara District Council was selected based on review of routine immunization performance in 2014 and 2015 by the Tanzania Ministry of Health Community Development, Gender, Elderly, and Children. The report described several regions that did not reach the ≥95% coverage MR2 targets, including Dar-es-Salaam, Lindi, Mtwara, Njombe, Coastal, Tanga, Morogoro, and Mwanza regions. Mtwara region was randomly selected from this list, after which Mtwara District Council was randomly selected from the seven regional councils as the study area. This area is inhabited by peasants and petty business [2] and had 28 health facilities providing vaccination services.

### Study design

A cross-sectional community-based study was conducted in which 1,000 mothers of children born during January 2014 to January 2015 were interviewed and vaccination cards for children were reviewed. This study was undertaken during January - April 2017. Participants had to be residents of the study area for at least the past three years and have at least one child born during the period of interest. We employed multistage cluster sampling to select 10 of the 21 area wards and five villages from each ward. Sample size per village was calculated proportionate to population size of the selected 50 villages. Bottle spinning was employed to select the starting household from which a mother/guardian was selected for interview. If the household did not have a mother of a child born during January 2014 to January 2015, the nearest neighbor household was used to replace that participant. Data were collected from guardians/mothers using a pre-tested semi-structured questionnaire administered at home. If the household had more than one eligible child, the parent/guardian responded for an older child. Information collected included sociodemographic characteristics, awareness of the MR2 vaccine, vaccination status of the child, and characteristics about the vaccination health facility used by the family. Child vaccination cards were examined to assess the vaccination status of the children.

### Data management and analysis

Data were entered into EpiInfo version 3.5.1. Categorical variables were summarised in proportions. Differences between proportions were examined using chi-square test. For continuous variables, medians and interquartile ranges (IQR) were calculated. Bivariate and multivariate analyses were conducted to identify independent factors associated with non-uptake of MR2. Odds Ratios (OR) and adjusted Odds Ratios (aOR) and 95% Confidence Intervals (CI) were calculated. All analyses were two-tailed and the significance level was set at 5%.

## Results

### Socio-demographic characteristics of mothers and children recruited in the study

A total of 1,000 mothers of children aged <5 years were interviewed. The median age of children was 2.50 years [IQR, 2.0-3.3 years] and about half (53.5%) were males. Of the 1,000 recruited children, 972 (97.2%) were born at a health facility. All respondents were mothers of the recruited children. The median age of mothers was 26 (IQR, 22-30) years. Nearly all respondents (94.5%) had either primary education or no formal education. The majority of mothers (82.7%) were married and the principal occupational activity was “informal employment” (91.0%) (Table 1). Proportion of measles-rubella second dose (MR2) non-uptake by card verification. Of 1,000 children with vaccination cards reviewed, 558 (55.8%) did not receive MR2. Ndumbwe and Mpapura wards, neighboring wards which share the same health facility as well as the same vaccination post, had the highest proportions of unvaccinated children (81.0% and 72.2%, respectively) (Table 2).

**Table 1:** Socio-demographic of mothers and their children recruited in the study, Mtwara district council, Tanzania, 2017

Characteristics	Number	Percentage (%)
**Child’s age groups (yrs)**		
2.00-2.49	493	49.3
2.50-2.99	288	28.8
3.00-3.49	219	21.9
**Sex of children available on RCH card**		
Female	465	46.5
Male	535	53.5
**Child’s place of delivery**		
At health facility	972	97.2
At home	28	2.8
**Mother’s/caretaker age groups (yrs)**		
16-20	139	13.9
21-25	319	31.9
26-30	310	31.0
31-35	134	13.4
36+years	98	9.8
**Level of education**		
No formal education	494	49.4
Primary, but not completed	218	21.8
Primary, completed	233	23.3
Secondary, but not completed	26	2.6
Secondary, completed	28	2.8
Tertiary	1	0.1
**Marital status**		
Married	827	82.7
Single	77	7.7
Divorced	86	8.6
Widowed	7	0.7
Co-habiting	3	0.3
**Occupation**		
Formal employment	9	0.9
Housewife	57	5.7
Informal employment	910	91.0
Student	1	0.1
Unemployed	23	2.3

**Table 2:** Proportion of measles-rubella second dose (MR2) non-uptake by card verification

Ward	Unvaccinated	%	Vaccinated	%
KITERE	18	21.4	66	78.6
LIBOBE	60	60.6	39	39.4
MADIMBA	57	65.5	30	34.5
MAYANGA	41	41.0	59	59.0
MOMA	66	64.7	36	35.3
MPAPURA	78	72.2	30	27.8
MSANGAMKUU	47	47.0	53	53.0
MSIMBATI	39	42.9	52	57.1
NDUMBWE	81	81.0	19	19.0
ZIWANI	71	55.0	58	45.0
**TOTAL**	**558**	**55.8**	**442**	**44.2**

### Factors associated with non-uptake of MR2 Vaccine

Mothers who had completed primary education or no formal education were more likely than mothers with secondary or higher education to not have taken their children for MR2 vaccination (OR = 2.1; 95%CI 1.2-3.7; p<0.01). Other factors which were associated with MR2 non-uptake in bivariate analyses included living in a household with >5 people (OR = 1.8; 95%CI 1.4-2.4; p<0.001) and the mother being unaware of the age at which MR1 and MR2 should be administered (OR = 4.6; 95%CI 3.4-6.1; p<0.001). In multivariable analysis, significant factors that remained associated with non-uptake of MR2 included being unaware of the age at which MR1 and MR2 should be administered [aOR=3.50; 95%CI 1.98-6.21; p<0.001], having MR2 vaccination services offered at the local vaccination station fewer than three days per week [aOR=1.50; 95%CI 1.42-5.59; p<0.001], not having the MR2 vaccine available during vaccination days [aOR=3.38; 95%CI 1.08-10.61; p<0.01], visiting vaccine clinic on a poorly-attended day, leading to unwillingness of health workers to open the 10-dose vaccine vials for single child [aOR=3.80; 95% CI 2.12-6.79; p<0.001], and long waiting times for vaccination services [aOR=1.80; 95% CI 1.08-3.00; p<0.01] (Table 3).

**Table 3:** Independent factors associated with non-uptake of MR2 among children under five in Mtwara District Council

Variable	Number (%)	OR	95%CI	aOR	95%CI
**Mothers’ education level**					
No formal/primary	945(94.5)	2.1*	1.2-3.7	1.62-*	0.9-3.0
Secondary/Tertiary	55(5.5)		1		1
**Household size**					
Yes (>5)	330(33.0)	1.8**	1.4-2.4	1.3-*	0.7-2.3
No (≤5)	670(67.0)		1		1
**Awareness of ages at which MR1 and MR2 administered**					
Unaware	694(69.4)	4.6**	3.4–6.1	3.5**	2.0–6.2
Aware	306(30.6)		1		1
**Days health facility opened per week**					
<3	796(79.6)	1.5*	1.1- 2.1	1.5*	1.4-5.6
>3	204(20.4)		1		1
**Healthcare worker absence during vaccination days**					
Yes	120(12.0)	2.0*	1.3-3.0	1.4-*	0.5-3.7
No	880(88.0)		1		1
**Vaccine unavailability on vaccination day**					
Yes	875(87.5)	24.6**	11.9-51.3	3.4*	1.1-10.6
No	125(12.5)				
**Number of children available per session**					
<5 children	504(50.4)	3.3**	2.6- 4.3	3.8**	2.1-6.8
≥5 children	496(49.6)		1		1
**Waiting time for seeking vaccination services**					
>2 hours	434(43.4)	1.8**	1.4–2.4	1.8***	1.1 –3.0
≤ 2 hours	566(56.6)		1		1
**No healthcare reminder on MR2 during postnatal**					
Yes	339(33.9)	31.9**	19.3-52.6	1.8-*	0.8-4.0
No	661(66.1)		1		1

OR = Odds ratio, aOR = Adjusted odds ratio, % = percentage, 1 = reference variable C. I. = Coefficient interval.

* = p< 0.01

** = p< 0.001

*** = p < 0.05 and

-* = p ≥ 0.05.

## Discussion

We investigated factors associated with non-uptake of the second dose of measles-rubella vaccine in Mtwara district council of Tanzania, a district experiencing very poor coverage. We found that more than half of eligible children in the area were unvaccinated, falling short of the 95% coverage target for elimination of measles worldwide [3]. Although Tanzania has not experienced any reported measles outbreaks in recent years, the high proportion of unvaccinated children with MR2 poses a risk to individual protection and threatens the herd immunity necessary for community protection.

In Mtwara District Council, nearly 70% of unvaccinated children had caretakers who were unaware of the age at which MR1 and MR2 should be administered. This results are consistent with other studies where unawareness of the immunization schedule was significantly associated with poor uptake [4-6]. This suggests poor communication between healthcare staff and the community about the appropriate ages for vaccination. To improve coverage, each visit to the clinic including visits not specifically for vaccination should be seen as an opportunity to educate, and to schedule the next vaccine visit. In addition, even when a child is not visiting explicitly for the purposes of vaccination, children in the appropriate age groups should be thoroughly screened for vaccination status and vaccinated whenever possible. Children having their MR2 vaccination services offered at the local vaccination station fewer than three days per week were two times as likely to be unvaccinated with MR2. Sensitization of the community by healthcare workers and community leaders one day before vaccination days may be used as a strategy to improve and increase the number of eligible children who attend the clinic on the day of vaccination.

In this study, we found that unavailability of MR vaccine during scheduled vaccination days was significantly associated with non-uptake of MR2 among children in the community. This finding is consistent with previous studies [7-10]. This may be most easily addressed through timely distribution of MR vaccine by district immunization and vaccine officers, to ensure availability of vaccines at health facilities. Attending clinic on scheduled vaccination days when there were fewer than five children attending increased the odds of MR2 non-vaccination three times as much as attending when there were at least five children. This was associated with unwillingness of health workers to open the ten-dose vaccine vials when they could not use at least half of it. This is consistent with findings by others [6]. To avert this, we recommend use of village healthcare workers, village political leaders as well as religious leaders to be sensitized to encourage villagers to have large turnouts (attendance) on vaccination days. At a national and global level, to avoid wastage, we suggest communication with vaccine manufacturers to produce five-dose vials of MR vaccine. Although this might be more costly to the country in terms of transportation and storage space, it has the potential to improve vaccination coverage with MR2. Long waiting times for vaccination services was found to be associated with non-uptake of MR2. Waiting >2 hours increased the odds of non-vaccination by twofold, compared with waiting time of two hours or less. This probably was due to lack of community involvement in scheduling time to start vaccination services at respective health facilities. Similar findings had been observed in other studies conducted elsewhere [9,11]. To minimize this situation, and taking into account the importance of vaccination in Tanzania, healthcare workers at health facilities need to consider prioritizing vaccination services before attending other attending patients during vaccination days. The cross-sectional nature of this study limits the causal-effect interpretation of the independent factors identified to be associated with non-uptake of MR2. However, most of these factors have been reported in other studies indicating a consistency nature of findings, an important aspect of conclusion of causation.

## Conclusion

The measles-rubella second dose vaccination coverage in Mtwara District Council in 2017 was poor. Intervention measures should focus on community education and sensitization about MR2 vaccine as well as ensuring vaccine and services availability in all facilities at all times.

### What is known about this topic

- Unawareness of immunization schedule significantly associated with non-uptake of MR2 vaccine;
- Mother's level of education significantly associated with vaccine uptake although in this work the variable was not statistically significant when confounders were controlled.

### What this study adds

- Attending clinic on scheduled vaccination days when there were fewer than five children attending increased the odds of MR2 non-vaccination three times as much as attending when there were at least five children. This study suggests that to avoid wastage, we suggest communication with vaccine manufacturers to produce five-dose vials of MR vaccine. Although this might be more costly to the country in terms of transportation and storage space, it has the potential to improve vaccination coverage with MR2;
- Long waiting times for vaccination services was found to be associated with non-uptake of MR2. This calls for community sensitization and screening for vaccination status of children when visiting the health facility.

## Competing interests

The authors declare no competing interests.
